# Sodium Accumulation and Blood Capillary Rarefaction in the Skin Predispose Spontaneously Hypertensive Rats to Salt Sensitive Hypertension

**DOI:** 10.3390/biomedicines10020376

**Published:** 2022-02-04

**Authors:** Jan Šilhavý, Petr Mlejnek, Miroslava Šimáková, František Liška, Jan Kubovčiak, Eva Sticová, Michal Pravenec

**Affiliations:** 1 Institute of Physiology, Czech Academy of Sciences, 14220 Prague, Czech Republic; Petr.Mlejnek@fgu.cas.cz (P.M.); Miroslava.Simakova@fgu.cas.cz (M.Š.); Frantisek.Liska@lf1.cuni.cz (F.L.); Michal.Pravenec@fgu.cas.cz (M.P.); 2 Institute of Biology and Medical Genetics, First Faculty of Medicine, Charles University, General University Hospital, 12800 Prague, Czech Republic; 3 Laboratory of Genomics and Bioinformatics, Institute of Molecular Genetics, Czech Academy of Sciences, 14220 Prague, Czech Republic; kubovcij@img.cas.cz; 4 Institute for Clinical and Experimental Medicine, 14021 Prague, Czech Republic; evsc@ikem.cz; 5 Department of Pathology, Third Faculty of Medicine, Charles University, 10000 Prague, Czech Republic

**Keywords:** salt-sensitive hypertension, spontaneously hypertensive rat, skin, sodium, salt, blood pressure, capillary rarefaction, gene expression

## Abstract

Recent studies in humans and rats suggested that increased Na^+^ storage in the skin without parallel water retention may predispose to salt-sensitive hypertension. In the current studies, we compared tissue Na^+^ storage in salt sensitive spontaneously hypertensive rats (SHR) versus salt resistant normotensive Brown Norway (BN-*Lx*) rats. After salt loading (10 days drinking 1% NaCl solution), the SHR showed significant parallel increase in Na^+^-to-water as well as (Na^+^+K^+^)-to-water ratios suggesting increased storage of osmotically inactive Na^+^ in the skin while no significant changes in skin electrolyte concentrations were observed in BN-*Lx* rats. SHR rats after salt treatment exhibited a nonsignificant decrease in skin blood capillary number (rarefaction) while BN-*Lx* rats showed significantly increased skin blood capillary density. Analysis of dermal gene expression profiles in BN-*Lx* rats after salt treatment showed significant up-regulation of genes involved in angiogenesis and proliferation of endothelial cells contrary to the SHR. Since the skin harbors most of the body’s resistance vessels it is possible that blood capillary rarefaction may lead to increased peripheral resistance and salt sensitivity in the SHR.

## 1. Introduction

It has long been recognized that in some people substantially increasing dietary intake of salt increases blood pressure, whereas in others, increased salt intake has little or no effect on blood pressure. Although the blood pressure response to salt is a continuous variable and the trait of salt sensitivity, such as that of hypertension, is arbitrarily defined, it has been estimated that 30% to 50% of hypertensive humans are salt sensitive and approximately 25% of normotensive humans are salt sensitive [1,2]. Salt sensitivity confers an increased risk for the occurrence of hypertension and cardiovascular disease. Furthermore, pathophysiological mechanisms mediating salt sensitivity may contribute to the risk for cardiovascular disease beyond their effects on blood pressure [3]. Accordingly, the mechanisms of salt sensitivity continue to be studied intensively with the hope that better understanding of those mechanisms could lead to improved approaches to the prevention and treatment of salt-induced increases in blood pressure and cardiovascular disease.

Recent studies in humans and rats provided evidence that Na^+^ could be stored in the body, mainly in the skin, without a parallel water retention to buffer free extracellular Na^+^ [4,5]. Some investigators have proposed that accumulation of Na^+^ in skin tissue may influence blood pressure responses to changes in salt intake [6]. However, it is not known how Na^+^ accumulation in the skin predisposes to salt-sensitive hypertension. It is possible that the amount of Na^+^ stored in skin tissue may regulate blood pressure by influencing the extent to which a high salt intake affects blood volume and cardiac output, and vascular resistance, or both. For example, it is possible that increased accumulation of Na^+^ in skin may affect the skin microcirculation and reduce skin blood capillary density. Skin blood capillary rarefaction, the reduction in the density of capillaries, has been associated with hypertension [7,8,9]. Capillary rarefaction is believed to mediate blood pressure changes by altering peripheral vascular resistance. He et al. [10] showed that in hypertensive humans, a modest reduction in salt intake increased dermal capillary density. The mechanisms whereby salt causes changes in the skin microcirculation remain unclear. The spontaneously hypertensive rats (SHR) are salt sensitive when compared to Brown Norway (BN-*Lx*) rats [11], however, the role of tissue Na^+^ accumulation in the pathogenesis of salt sensitivity was not analyzed in this animal model. In the current study, we tested the hypothesis that Na^+^ accumulation in the skin may lead to blood capillary rarefaction and increased peripheral resistance and salt sensitivity in the SHR. In addition, we performed gene expression profiling in the skin to search for genes and pathways regulating dermal Na^+^ accumulation and angiogenesis.

## 2. Materials and Methods

### 2.1. Animals

We used 10-week-old males from SHR/OlaIpcv and BN-*Lx*/Cub strains (hereafter referred to as SHR and BN-*Lx*). The SHR/OlaIpcv (Rat Genome Database ID 631848) and BN-*Lx*/Cub (Rat Genome Database ID 61113) strains were obtained from the animal facility of the Institute of Physiology, Czech Academy of Sciences. The SHR/OlaIpcv strain was transferred to the Institute of Physiology from Olac, Ltd. Bicester, UK and is a substrain of the SHR/N inbred strain from NIH. BN-*Lx*/Cub is a substrain of the originally sequenced BN/NHsdMcwi strain (RGD ID 61498). In addition, it is a congenic strain carrying a segment of chromosome 8 from the PD/Cub (Polydactylous) strain with the *Lx* (Polydactly luxate syndrome) mutation, coded by the mutatnt *Zbtb16* gene [12,13]. We used young adult rats because our earlier experiments showed that at this age BN-*Lx* and SHR strains clearly differ in their blood pressure and salt sensitivity [11]. Rats had free access to standard diet (Sniff^®^ R-Z, Soest, Germany) containing 0.25% Na^+^ and water and they were held under humidity- and temperature-controlled conditions on a 12–12-h light-dark cycle. All animal experiments were conducted in compliance with the Animal Protection Law of the Czech Republic and were approved by the Ethics Committee of the Institute of Physiology, Czech Academy of Sciences, Prague (protocol code 71/2015).

### 2.2. Experimental Protocol

There were 4 experimental groups: Group 1—BN-*Lx* (N = 6) on regular chow and tap water; Group 2—BN-*Lx* (N = 6) on regular chow and 1% NaCl solution; Group 3—SHR (N = 6) on regular chow and tap water and Group 4—SHR (N = 6) on regular chow and 1% NaCl solution. On day 1, rats were placed into metabolic cages with standard chow and tap water. Every day 24-h aliquots of urine for Na^+^ and Cl^−^ were collected and fluid intake was recorded. On day 4, Group 2 (BN-*Lx*) and 4 (SHR) rats were given free access to 1% NaCl solution instead of tap water. On day 15, blood samples (for Na^+^, K^+^, and Cl^−^ measurements) were taken from tail vein before the animals were killed by cervical dislocation. Samples of skin (1 cm × 2 cm) were collected from the middle of subscapular region for determination of gene expression profiles (snap frozen in liquid nitrogen and stored at −80 °C) and histological examination (stored in 4% formalin solution).

### 2.3. Tissue Electrolyte Determination

Intestines and stomach were completely removed from rats to exclude remains of chow. The skins, gastrocnemius muscles, and carcasses (body without skin, intestines and stomach) were weighed to determined wet weight (WW) and then desiccated at 105 °C for 36 h to determine dry weight (DW). The difference between WW and DW is considered as tissue water content. Ashing of carcasses was performed at 200 °C and 450 °C for 20 h at each temperature level, and the bones were sieved from the carcass ashes. Ashing of skins was performed at 200 °C and 450 °C for 10 h at each temperature level. Ashes were sent for analysis of Na^+^ and K^+^ concentrations by atomic adsorption spectrometry to ALS Czech Republic, Ltd. (Prague, Czech Republic). Cl^−^ concentrations in the ashes were measured by titration with 0.1 N silver nitrate in the State Veterinary Institute (Prague, Czech Republic). Since the body weights of SHR and BN-*Lx* strains are significantly different, all chemical parameters in tissues are expressed as relative values corrected to dry weights and in the case of relative water content to wet weights. We also calculated Na^+^-to-water, K^+^-to-water, and (Na^+^+K^+^)-to-water ratios. Parallel increases in skin Na^+^-to-water and skin (Na^+^+K^+^)-to-water ratios indicate increased Na^+^ cation abundance relative to water and hence osmotically inactive Na^+^ storage in the skin.

### 2.4. Gene Expression Profiling

We used RNAseq method for determination of gene expression profiles in the skin as described [14]. Library preparation was carried out with SENSE total RNA AEQ library prep kit for Illumina (PN A01107, Lexogene GmbH, Vienna, Austria). Library size distribution was evaluated on the Agilent 2100 Bioanalyzer using the High Sensitivity DNA Kit (Agilent Technologies Inc, Santa Clara, CA, USA). Libraries were sequenced on the Illumina NextSeq^®^ 500 instrument using 84 bp single-end configuration. Read quality was assessed by FastQC. For subsequent read processing, a bioinformatic pipeline nf-core/rnaseq version 1.4.2, was used. Individual steps included removing sequencing adaptors with Trim Galore, mapping to reference genome Rnor_6.0 (Ensembl annotation version 99) with HISAT2 and quantifying expression on gene level with featureCounts. Per gene mapped counts served as input for differential expression analysis using DESeq2 R Bioconductor package. Prior to the analysis, genes not expressed in at least two samples were discarded. Shrunken log2-fold changes using the adaptive shrinkage estimator were used for differential expression analysis. We supplied experimental model assuming sample treatment as main effect. Genes exhibiting minimal absolute log_2_-fold change value of 1 and statistical significance (adjusted *p*-value < 0.05) between compared groups of samples were considered as differentially expressed (Figure S1). Gene set enrichment analysis was carried out using gene length bias aware algorithm implemented in goseq R Bioconductor package with KEGG pathways and GO terms data.

### 2.5. Number of Blood and Lymph Capillaries

Two full-thickness skin excisions taken from each animal were fixed in 4% formaldehyde and routinely processed by paraffin technique. Tissue sections 4 μm thick were cut from each block and stained with hematoxylin and eosin (H&E).

For immunohistochemical analysis, tissue sections (4 μm-thick) were pre-treated by heat mediated antigen retrieval and incubated with primary rabbit polyclonal anti-CD31/PECAM-1 antibody (AP31323PU-N, Acris Antibodies GmbH, Herford, Germany, dilution 1:1000) to detect vascular endothelia, and with primary mouse monoclonal antibody anti-Podoplanin (clone LF3/B7/D5B27, NB110-96423, Novus Biologicals, Cambridge, UK, dilution 1:200) to detect lymphatic vessels. The primary antibodies were applied overnight at 4 °C. The CD31 antibody was detected by biotinylated goat anti-rabbit IgG (H + L) (BA-1000-1.5, Vector Laboratories, Burlingame, CA, USA, dilution 1:200), after which the sections were incubated with VECTASTAIN^®^ Elite ABC-HRP Reagent, Peroxidase, R.T.U. (PK-7100, Vector Laboratories) for 30 min. The Simple Stain MAX PO (MULTI) Universal Immuno-peroxidase Polymer anti-mouse, anti-rabbit Histofine (414152F, Nichirei Biosciences, Tokyo, Japan) was used to detect the primary anti-podoplanin antibody. Finally, the sections were stained with the Dako Liquid DAB Substrate-Chromogen System (K3468, Dako, Glostrup, Denmark) and counterstained with Harris’s haematoxylin.

For quantifications, 10 non-overlapping consecutive fields at magnification ×40 with representation of full-thickness dermal compartment were captured from each tissue section using a microscope Olympus BX51 and a camera MICROPIX. Positively stained dermal blood vessels and lymphatic vessels were counted in each field using Micropix v.2.0.4.0 image processing software (Micropix, Ltd., Chichester, UK). The results were expressed as the mean microvessel count with standard deviation.

### 2.6. Statistical Analysis

Results are expressed as means ± S.E.M. SigmaPlot^®^ 12 software package was used for two-way ANOVA to test for strain x salt interactions. For variables showing evidence of strain x salt interaction effects, we used Holm Sidak testing that adjusts for multiple comparisons to determine whether the effects of salt were significant in SHR versus BN-*Lx* rats.

## 3. Results

### 3.1. Tissue Electrolyte Concentrations and Water Content

Table 1 shows the body weights, tissue water content and electrolyte concentrations in all four experimental groups of rats. The SHR rats were significantly heavier when compared to BN-*Lx* rats and therefore all electrolyte concentrations in tissues are expressed as relative values corrected to dry weights and in the case of relative water content to wet weights. Total body Na^+^ significantly increased after salt loading in the SHR while no difference was observed between BN-*Lx* control versus BN-*Lx* salt-treated rats. There were no significant differences in total body K^+^ between strains and treatment. On the other hand, when rats were treated with salt, the SHR showed significantly higher total body Cl^−^ levels while BN-*Lx* rats showed no significant difference. Salt treatment had no effects on relative total body water (Table 1).

**Figure 1:**
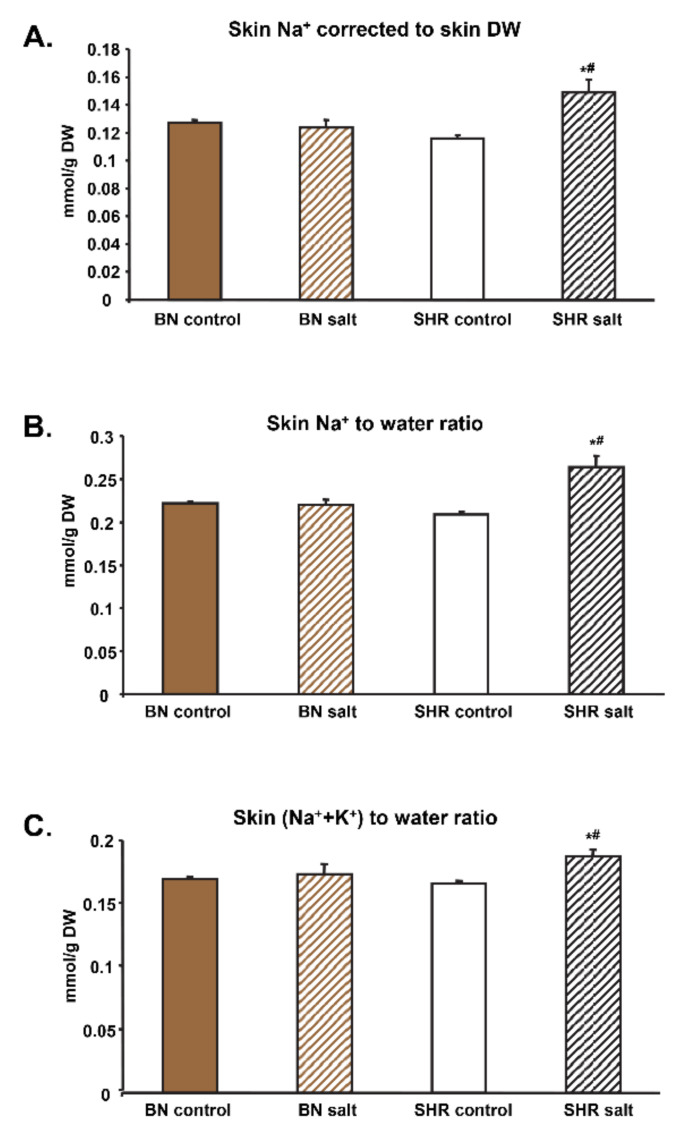
Electrolyte concentrations in the skin of SHR and BN-*Lx* strains. (**A**) Salt treatment of SHR rats was associated with significant accumulation of relative (corrected to dry weight) Na^+^ in the skin. (**B**) SHR rats treated with salt exhibited significantly increased Na^+^-to-water ratio (**B**) and also (Na^+^+K^+^)-to-water ratio (**C**). Parallel increase of Na^+^-to-water ratio and (Na^+^+K^+^)-to-water ratio indicates higher amount of osmotically inactive Na^+^ storage. * denotes two-way ANOVA significant strain × salt interaction effects (*p* < 0.05), ^#^ denotes significantly higher value in the SHR after salt treatment (*p* < 0.05).

Skin Na^+^ increased after salt treatment in the SHR but not in BN-*Lx* rats (Figure 1A, Table 1). There were no significant differences in skin K^+^ concentrations both between strains and salt treatment. After treatment with salt, SHR rats showed significantly higher Cl^−^ levels in the skin while BN-*Lx* rats showed no significant difference. Skin was the major depot for Na^+^ accumulation when skin of BN-*Lx* and SHR rats contained 32% and 42% of total body Na^+^, respectively.

Carcass Na^+^ increased in SHR rats after salt treatment but not in BN-*Lx* rats. Carcass K^+^ levels in BN-*Lx* rats were not significantly different from the SHR. On the other hand, Cl^−^ carcass concentrations increased in the SHR after salt treatment while no significant differences were observed between BN-*Lx* control and experimental groups (Table 1).

No significant differences in muscle electrolyte concentrations and water content were observed between strains and salt treatment (Table 1).

No significant differences were observed in bone Na^+^ concentrations. On the other hand, BN-*Lx* rats showed significantly increased bone K^+^ after salt treatment and had higher K^+^ levels when compared to SHR rats. There were small but significant differences in bone Cl^−^ concentrations among the groups (Table 1).

### 3.2. Na^+^-to-Water, K^+^-to-Water and (Na^+^+K^+^)-to-Water Ratios

Table 2 shows the relationships between Na^+^, K^+^, and the sum of the two cations (total effective osmolytes) with total body water. The ratio of total body Na^+^ per total body water was increased by salt only in the SHR while remained unchanged in BN-*Lx* rats. This increased ratio of total body Na^+^-to-water was mainly due to higher storage of Na^+^ in the skin of SHR rats because no significant differences were observed in carcass, muscle and bone Na^+^-to-water ratios (Figure 1B, Table 2).

Total body K^+^-to-water ratio was higher in BN-*Lx* rats when compared to SHR rats on high salt intake. Carcass K^+^-to-water ratios was reduced while skin and bone K^+^-to-water ratios after salt were increased in BN-*Lx* rats but not in SHR rats (Table 2).

Salt treatment increased total body (Na^+^+K^+^)-to-water ratio in SHR rats but not in BN-*Lx* rats which was mainly due to significant increase in skin (Na^+^+K^+^)-to-water ratio when compared to BN-*Lx* rats (Figure 1C). On the other hand, bone (Na^+^+K^+^)-to-water ratio was increased after salt in BN-*Lx* rats but not in SHR rats. No significant differences in muscle (Na^+^+K^+^)-to-water ratio were observed between strains and salt treatment (Table 2). Parallel increases in Na^+^-to-water and (Na^+^+K^+^)-to-water ratios indicate increased Na^+^ cation abundance relative to water and hence osmotically inactive Na^+^ storage in the skin of SHR rats.

### 3.3. Fluid Ingestion and Cumulative Na^+^ Balance

**Figure 2:**
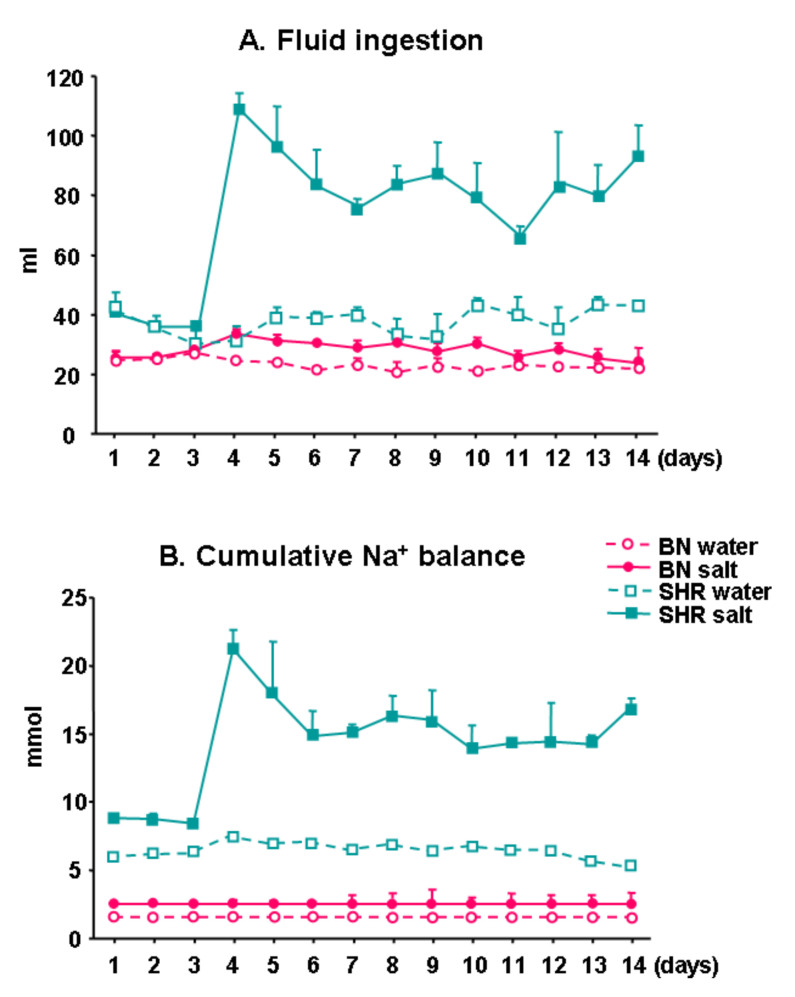
Fluid ingestion (**A**) and cumulative Na^+^ balance (**B**). On day 4, experimental groups of BN-*Lx* and SHR rats were given free access to 1% NaCl solution while control BN-*Lx* and SHR stayed on tap water. The SHR rats ingested significantly more NaCl solution when compared to BN-*Lx* rats and exhibited significantly increased cumulative Na^+^ balance.

Figure 2 shows fluid ingestion and cumulative Na^+^ balance in SHR and BN-*Lx* rats drinking ad libitum either tap water or salt solution. As can be seen, on high salt diet the SHR ingested significantly more salt solution and exhibited significantly higher cumulative Na^+^ balance when compared to BN-*Lx* rats. These differences remained significant when the ingested fluid was corrected to body weight (data not shown). These observations suggest that increased Na^+^ accumulation in the skin of SHR rats might be due to increased salt ingestion.

### 3.4. Skin Blood and Lymph Capillary Density

**Figure 3:**
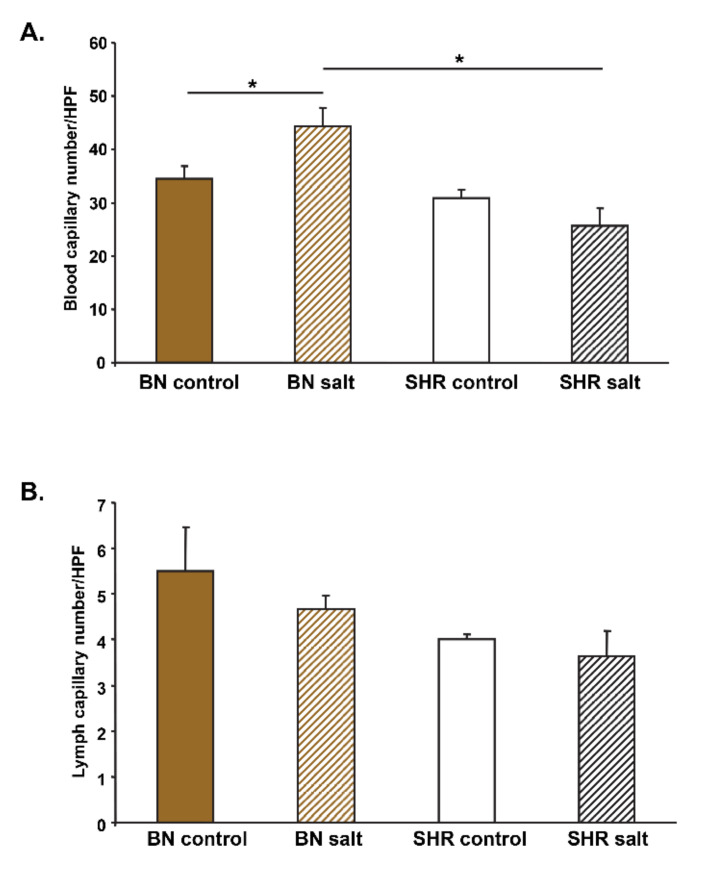
Skin blood and lymph capillary number in BN-*Lx* and SHR rats drinking either tap water (controls) or 1% NaCl solution (salt) for 10 days. (**A**) Two-way ANOVA showed significant strain × salt interaction effects (*p* = 0.026). BN-*Lx* rats exhibited significantly increased blood capillary number after salt treatment when compared to BN-*Lx* controls and to SHR treated with salt. (**B**) No significant differences by two-way ANOVA were observed in lymph capillary density between strains and treatments. * denotes *p* < 0.05.

**Figure 4:**
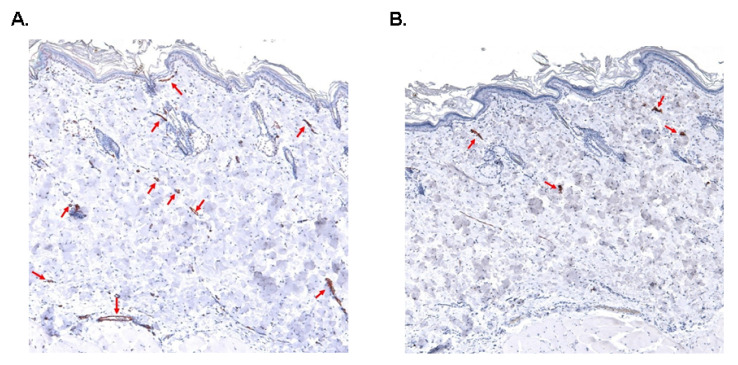
Representative images of skin blood and lymphatic capillaries. (**A**). Anti-CD31/PECAM-1 immunohistochemistry. The antibody stains endothelial lining of dermal blood vessels (arrows). Original magnification ×40. (**B**). Anti-podoplanin immunohistochemistry. Primary antibody highlights dermal lymphatic vessels (arrows). Original magnification ×40.

We measured number of blood capillaries in the skin of BN-*Lx* and SHR rats on normal or high salt intake. Two-way ANOVA showed significant strain x salt interaction effects on blood capillary number in the skin. As can be seen in Figure 3A, BN-*Lx* rats after salt treatment exhibited significantly increased skin blood capillary number contrary to SHR rats. On the hand, we observed no significant differences in the number of lymph capillaries between BN-*Lx* and SHR strains and between control and high salt intake (Figure 3B). Figure 4 shows representative histological images of blood and lymphatic capillaries.

### 3.5. Gene Expression Profiles in the Skin

To search for molecular mechanisms responsible for differences in Na^+^ accumulation in the skin and salt-dependent hypertension, we performed analysis of gene expression profiles using RNAseq method in response to salt loading in BN-*Lx* and SHR strains (Table 3 and Table 4). Gene set enrichment analysis (GSEA) on biological processes (BP) in BN-*Lx* rats identified as the most significantly up-regulated genes involved in regulation of angiogenesis and proliferation of endothelial cells (Table 3). On the other hand, in the SHR after salt treatment, GSEA on BP identified as the most significantly up-regulated genes involved mainly in regulation of keratinocyte differentiation and epidermis development, negative regulation of peptidase activity and innate immunity (Table 4).

## 4. Discussion

In the current study, we found that salt sensitive SHR rats after salt loading accumulated significantly higher content of osmotically inactive Na^+^ mainly in the skin and exhibited skin capillary rarefaction when compared to normotensive salt resistant BN-*Lx* rats. Recent paper by Rossitto et al. [15] reported that salt loading (1% NaCl solution for 3 weeks) of SHRSP (SHR stroke-prone) rats resulted in parallel increase in skin Na^+^ and water accumulation while no such increases were observed in normotensive salt resistant WKY rats. This result is different from previously published findings by Titze et al. [4,16,17] who found significant accumulation of osmotically inactive Na^+^ in the skin of different models of salt sensitive hypertension. It also differs from results of the current study since we detected no significant differences in tissue water accumulation between SHR and BN-*Lx* rats and between treatment with tap water or 1% NaCl solution. Water content in the skin: BN-*Lx* control 55.8%, BN-*Lx* after salt 56.0%, SHR control 55.5%, SHR after salt 55.4%; water content in the carcass: BN-*Lx* control 68.8%, BN-*Lx* after salt 68.4%, SHR control 67.2%, SHR after salt 67.9% (calculated from data shown in Table 1). These differences could be explained by differences in measurements of tissue water and electrolyte concentrations. For estimation of Na^+^ and water content Rossitto et al. used relatively small skin samples and other tissues samples that were digested in HNO_3_, while we and Titze et al. [4,16,17] measured electrolyte and water content in the whole skin and carcass and used desiccation and ashing before electrolyte measurement. An additional explanation for the discrepant results could be related to differences in the rat strains studied.

The SHR rats ingested much more salt solution when compared to BN-*Lx* rats and exhibited significantly increased cumulative Na^+^ balance. These results suggest that increased Na^+^ accumulation in the skin of SHR rats might be due to increased salt ingestion. It has been reported that the SHR ingests significantly more salt when compared to normotensive WKY (Wistar Kyoto) rats. For instance, in the study by Chrysant et al. [18] the intake of 1% NaCl solution was almost 4 times higher when compared to WKY rats. Similar findings were reported by other investigators [19,20,21].

A number of studies reported blood capillary rarefaction in the established stage of hypertension in many animal models including the SHR and it was suggested that capillary rarefaction can contribute to increase of total peripheral resistance [22,23]. Experiments in rats have demonstrated that salt intake has a direct effect on microvascular circulation and may be independent of blood pressure. For example, a rapid loss of microvessels in cremaster muscle occurred in Sprague-Dawley rats with reduced renal mass and hypertension but also in normotensive sham operated controls on chronic (4–6-week) as well as acute (3 days) high salt diet [22,24]. It is possible that salt, rather than working through blood pressure, may have a direct effect on microvascular network and could be one of the underlying causes for the microvascular rarefaction that occurs in hypertension.

Skin capillary rarefaction was also found in humans with essential hypertension when compared to normotensive controls [25]. Although measurement of skin capillary density in humans was used as a surrogate for capillary density in other organs these results suggest that salt sensitive hypertension in humans is associated with blood capillary rarefaction in the skin. He et al. [10] reported that in hypertensive humans, a modest reduction in salt intake increased dermal capillary density. It was also suggested that capillary rarefaction was likely to be the primary vascular abnormality that is not secondary to high blood pressure [9].

BN-*Lx* rats treated with salt showed significantly increased blood capillary density in the skin which was associated with significantly increased expression of genes regulating angiogenesis including *Ereg* (epiregulin), *Vegfd* (vascular endothelial growth factor D), *Sfrp2* (secreted frizzle-related protein 2), *Angpt4* (angiopoietin 4) and other genes from Positive regulation of angiogenesis and Positive regulation of endothelial cell proliferation biological processes (Table 3). Keratinocytes are able to produce several complement components. The role of complement in angiogenesis has been reviewed recently [26], however, its role in regulating skin capillary density needs further studies. Additional genes with significantly increased expression after treatment of BN-*Lx* rats with salt might be implicated in angiogenesis, including *Ccl11*, *Ccr1* or *Fn1*.

On the other hand, SHR after salt loading showed no significant change in blood capillary density which was associated with increased dermal expression of genes regulating Keratinocyte differentiation, epidermis development and Negative regulation of peptidase activity including serpins (*Serpinb3a*, *Serpinb12*, *Serpina12*) which are known inhibitors of angiogenesis [27]. Osmotic stress is a potent inflammatory stimulus by triggering proinflammatory cytokine release and inflammation and also has antiangiogenic effects. During the inflammatory processes in the skin, keratinocytes act as immuno-modulators, managing inflammation via a rigorously coordinated network of inflammatory cascades [28]. Activated keratinocytes switch from their inactive status to a migratory, proliferative and pro-inflammatory phenotype and release cytokines (interleukins) and chemokines and chemokine receptors. As can be seen in Table 3 and Table 4, BN-*Lx* skin upon salt treatment expressed genes coding for CCL11 pro-angiogenic chemokine while the SHR expresses prolinflammatory IL-1b and IL-36g cytokines. There is considerable evidence implicating also *Serpinb3a* in skin inflammation [29].

In the current study, we observed no significant strain and salt effects on lymph capillary density in BN-*Lx* and SHR rats (Figure 2B). Machnik et al. [30] reported that treatment of Sprague-Dawley rats with 1% NaCl solution for drinking resulted in higher mean arterial pressure by 20 mmHg, in higher skin Na^+^ concentrations and increased lymph capillary but not blood capillary number in the ear skin. In another paper, Machnik et al. [31] reported that Sprague-Dawley rats were salt resistant (did not increased blood pressure after 1% NaCl solution for drinking) and exhibited significantly increased lymph capillary density in the ear skin after salt treatment. In addition, we observed no significant differences in dermal expression of *Vegfc*, *Nfat5* (TonEBP), *Tnfa* or *Nos3* (eNOS) genes between BN-*Lx* and SHR strains on normal and high salt intake. These genes were implicated in interstitial electrolyte clearance via TONEBP and VEGFC/VEGFR3-mediated modification of cutaneous lymphatic capillary function [32]. These discrepant results could be explained by differences in genetic backgrounds of SHR and BN-*Lx* versus Sprague-Dawley rats. In addition, we measured capillary density in the skin from the back of rats, not in the ear skin and blood flow might not be uniform over the entire skin surface [33].

**Figure 5:**
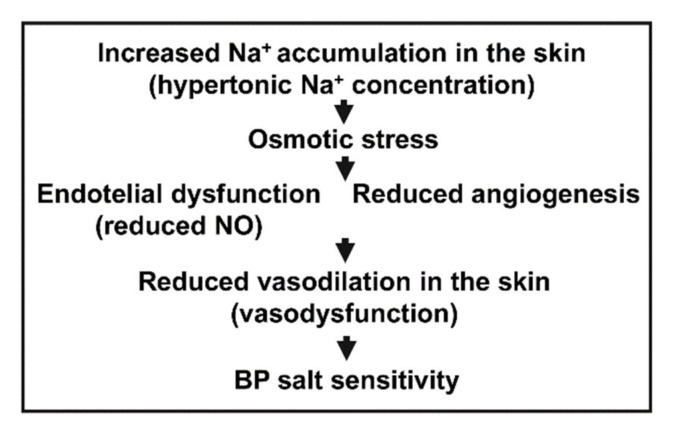
The proposed sequence of events showing the role of Na^+^ accumulation in the pathogenesis of salt sensitive hypertension. According to the vasodysfunction theory [35,36] the normal response to salt loading in salt resistant controls is vasodilation and a decrease in systemic vascular resistance. The failure to normally vasodilate and reduce systemic vascular resistance in the skin might be caused by skin capillary rarefaction and/or by endothelial dysfunction.

Results of the current study suggest a possible connection of skin Na^+^ accumulation with reduced angiogenesis in the pathogenesis of salt sensitive hypertension in the SHR. Hypertonic interstitial Na^+^ overload in the skin, which harbors most of the body’s resistance vessels, may lead to skin blood capillary rarefaction and endothelial dysfunction which together result in increased peripheral resistance and thus contribute to the pathogenesis of salt sensitivity (Figure 5). It is possible that both reduced vasodilatation (vasodysfunction) and capillary rarefaction in the SHR after salt loading could be determined by the same pathway, for instance by decreased availability of NO. NO is known to stimulate both vasorelaxation and angiogenesis. Hypothetically it might be possible that after salt loading the first response would be rapid NO induced vasorelaxation and opening of closed capillaries that would be followed by NO induced angiogenesis.

Significant differences between BN-*Lx* and SHR strains in salt sensitivity, skin Na^+^ accumulation and blood capillary density suggest strong genetic effects. The BXH/HXB recombinant inbred (RI) strains, derived from BN-*Lx* and SHR progenitors, show significant heritability of salt sensitivity of blood pressure [34]. Accordingly, testing the RI strains for salt effects on skin blood and lymph capillary density and on skin electrolyte concentration will enable linkage and correlation analyses with blood pressure salt sensitivity and thus identification of responsible pathophysiological mechanisms.

## 5. Conclusions

Results of the current study showed that salt-sensitive hypertension in the SHR is associated with increased storage of osmotically inactive Na^+^ in the skin and skin capillary rarefaction when compared to normotensive, salt-resistant BN-*Lx* rats. Since the skin harbors most of the body’s resistance vessels it is possible that blood capillary rarefaction may lead to increased peripheral resistance and thus represent new mechanism of salt sensitivity in the SHR.

## Figures and Tables

**Table 1:** Absolute wet weights and dry weights in various tissues and relative concentrations of Na^+^, K^+^, and Cl^−^ in tissues from BN-*Lx* and SHR rats given either tap water or 1% saline for drinking.

Traits	BN-*Lx* Control	BN-*Lx* Salt	SHR Control	SHR Salt	Source of Variation (*p*)		
					Strain	Salt	Strain × Salt Interaction
Body WW (skin WW + Carcass WW) (g)	228 ± 10	209 ± 4	308 ± 3	303 ± 2	<0.001	0.067	0.253
Body DW (g) (Carcass DW + Skin DW)	76 ± 3	71 ± 3	109 ± 1	106 ± 2	<0.001	0.102	0.664
Carcass WW (g)	186 ± 8	168 ± 3	235 ± 1	228 ± 2	<0.001	0.032	0.246
Carcass DW (g)	58 ± 2	53 ± 2	77 ± 1	74 ± 2	<0.001	0.050	0.571
Carcass water (g)	128 ± 6	115 ± 1	158 ± 1	155 ± 1	<0.001	0.036	0.168
Carcass relative water (mL/g WW)	0.687 ± 0.003	0.685 ± 0.007	0.674 ± 0.002	0.678 ± 0.004	0.046	0.788	0.438
Skin WW (g)	43 ± 1	41 ± 1	72 ± 2	74 ± 1	<0.001	0.964	0.428
Skin DW (g)	18 ± 2	18 ± 4	32 ± 4	32 ± 4	<0.001	0.752	0.986
Skin water (g)	24 ± 1	23 ± 1	40 ± 2	41 ± 1	<0.001	0.843	0.230
Skin relative water (mL/g WW)	0.571 ± 0.006	0.565 ± 0.009	0.552 ± 0.006	0.563 ± 0.009	0.204	0.770	0.310
rTBW (mL/g WW)	1.258 ± 0.009	1.250 ± 0.015	1.226 ± 0.005	1.241 ± 0.013	0.108	0.765	0.329
Bone ash (g)	7.6 ± 0.3	7.1 ± 0.1	8.6 ± 0.2	8.2 ± 0.1	<0.001	0.059	0.982
rTB Na^+^ (mmol/g DW)	0.220 ± 0.005	0.213 ± 0.011	0.192 ± 0.005	0.239 ± 0.011 ^#^	0.938	0.045	0.013
rTB K^+^ (mmol/g DW)	0.293 ± 0.010	0.277 ± 0.018	0.246 ± 0.007	0.263 ± 0.010	0.030	0.974	0.202
rTB Cl^−^ (mmol/g DW)	0.110 ± 0.027	0.072 ± 0.005	0.088 ± 0.011	0.123 ± 0.008 ^‡^	0.376	0.934	0.040
rCarcass Na^+^ (mmol/g DW)	0.093 ± 0.002	0.089 ± 0.006	0.077 ± 0.003 ^†^	0.090 ± 0.002 ^#^	0.070	0.220	0.036
rCarcass K^+^ (mmol/g DW)	0.197 ± 0.009	0.166 ± 0.009	0.158 ± 0.005	0.171 ± 0.004	0.026	0.726	0.091
rCarcass Cl^−^ (mmol/g DW)	0.015 ± 0.001	0.013 ± 0.001	0.015 ± 0.001	0.021 ± 0.001 ^#‡^	0.004	0.026	0.002
rSkin Na^+^ (mmol/g DW)	0.127 ± 0.002	0.124 ± 0.005	0.116 ± 0.002	0.149 ± 0.009 ^#‡^	0.268	0.025	0.012
rSkin K^+^ (mmol/g DW)	0.096 ± 0.001	0.100 ± 0.005	0.088 ± 0.002	0.092 ± 0.006	0.078	0.422	0.950
rSkin Cl^−^ (mmol/g DW)	0.070 ± 0.009	0.059 ± 0.004	0.073 ± 0.010	0.102 ± 0.007 ^‡^	0.370	0.978	0.037
rBone Na^+^ (mmol/g carcass DW)	0.068 ± 0.001	0.072 ± 0.001	0.064 ± 0.003	0.065 ± 0.001	0.173	0.775	0.156
rBone K^+^ (mmol/g carcass DW)	0.055 ± 0.004	0.070 ± 0.005	0.040 ± 0.003	0.038 ± 0.001	0.003	0.056	0.234
rBone Cl^−^ (mmol/g carcass DW)	0.019 ± 0.001	0.020 ± 0.001 *	0.018 ± 0.001 ^†^	0.019 ± 0.001 ^‡^	<0.001	0.103	0.046
Serum Na^+^ (mmol/L)	141.9 ± 0.3	143.2 ± 0.6	143.2 ± 0.6	143.4 ± 0.8	0.245	0.265	0.381
Serum K^+^ (mmol/L)	5.2 ± 0.1	5.0 ± 0.1	5.2 ± 0.1	4.9 ± 0.1 ^‡^	0.524	0.009	0.294
Serum Cl^−^ (mmol/L)	105.2 ± 0.8	106.6 ± 0.7	103.7 ± 0.7	105.2 ± 0.9	0.094	0.104	0.935

Two-way ANOVA results: *p* values of statistical significance for effects strain, salt treatment and strain x salt interaction (the effects of salt are different in BN-*Lx* versus SHR rats). For pairwise multiple comparison procedures Holm Sidak testing was used: * denotes significant *p* < 0.05 effects of salt within BN-*Lx* rats; ^#^ denotes significant *p* < 0.05 effects of salt within SHR rats; ^†^ denotes *p* < 0.05 significant differences between strains on normal salt intake; ^‡^ denotes *p* < 0.05 significant differences between strains on high salt intake.

**Table 2:** Na^+^-to-water, K^+^-to-water, and (Na^+^+K^+^)-to-water ratios.

Traits	BN-*Lx* Control	BN-*Lx* Salt	SHR Control	SHR Salt	Source of Variation (*p*)		
					Strain	Salt	Strain × Salt Interaction
Na^+^-to-water ratio, mmol/mL							
TB Na^+^/TB W	0.051 ± 0.001	0.050 ± 0.001	0.049 ± 0.002	0.058 ± 0.001 ^#‡^	0.032	0.005	0.002
Carcass Na^+^/Carcass W	0.042 ± 0.001	0.041 ± 0.001	0.037 ± 0.002 ^†^	0.043 ± 0.001 ^#^	0.229	0.132	0.017
Skin Na^+^/Skin W	0.095 ± 0.001	0.096 ± 0.001	0.094 ± 0.001	0.115 ± 0.004 ^#‡^	0.003	<0.001	0.001
Bone Na^+^/TB W	0.026 ± 0.001	0.028 ± 0.001	0.026 ± 0.002	0.024 ± 0.003	0.146	0.900	0.089
K^+^-to-water ratio, mmol/mL							
TB K^+^/TB W	0.087 ± 0.002	0.081 ± 0.003	0.073 ± 0.008 ^†^	0.079 ± 0.004	0.005	0.924	0.018
Carcass K^+^/Carcass W	0.090 ± 0.003	0.082 ± 0.004 *	0.074 ± 0.005 ^†^	0.081 ± 0.001	0.011	0.804	0.015
Skin K^+^/Skin W	0.073 ± 0.001	0.077 ± 0.002 *	0.071 ± 0.001	0.070 ± 0.002 ^‡^	0.010	0.337	0.046
Bone K^+^/TB W	0.020 ± 0.001	0.027 ± 0.003 *	0.016 ± 0.001	0.014 ± 0.001 ^‡^	<0.001	0.068	0.019
(Na^+^+K^+^)-to-water ratio, mmol/mL							
TB (Na^+^+K^+^)/TB W	0.138 ± 0.003	0.131 ± 0.004	0.124 ± 0.004 ^†^	0.137 ± 0.001 ^#^	0.293	0.397	0.016
Carcass (Na^+^+K^+^)/Carcass W	0.132 ± 0.004	0.122 ± 0.005	0.114 ± 0.005 ^†^	0.124 ± 0.001	0.064	0.958	0.035
Skin (Na^+^+K^+^)/Skin W	0.168 ± 0.002	0.172 ± 0.008	0.165 ± 0.002	0.186 ± 0.005 ^#‡^	0.085	0.002	0.018
Bone (Na^+^+K^+^)/TB W	0.046 ± 0.001	0.054 ± 0.003 *	0.042 ± 0.002	0.039 ± 0.001 ^‡^	0.002	0.226	0.025

Two-way ANOVA results: *p* values of statistical significance for effects strain, salt treatment and strain × salt interaction (the effects of salt are different in BN-*Lx* versus SHR rats). For pairwise multiple comparison procedures Holm Sidak testing was used: * denotes significant *p* < 0.05 effects of salt within BN-*Lx* rats; ^#^ denotes significant *p* < 0.05 effects of salt within SHR rats; ^†^ denotes *p* < 0.05 significant differences between strains on normal salt intake; ^‡^ denotes *p* < 0.05 significant differences between strains on high salt intake.

**Table 3:** Differentially expressed genes from biological processes (BP) identified by Gene set enrichment analysis (GSEA) in BN-*Lx* rats treated with 1% NaCl drinking solution versus control BN-*Lx* rats drinking tap water.

Symbol	Name	logFC	Adjusted *p* Value
**BP: Positive regulation of angiogenesis, *p* = 8.7 × 10^−7^**			
*Ereg*	epiregulin	3.39	0.0018
*Vegfd*	vascular endothelial growth factor D	1.60	0.0075
*Angpt4*	angiopoietin 4	2.86	0.0018
*Ccl11*	C-C motif chemokine ligand 11	2.06	0.0067
*Sfrp2*	secreted frizzled-related protein 2	3.80	0.00015
*F3*	coagulation factor III, tissue factor	2.04	0.000027
*C6*	complement C6	2.05	0.0073
**BP: Positive regulation of endothelial cell proliferation, *p* = 1.30 × 10^−4^**			
*Vegfd*	vascular endothelial growth factor D	1.60	0.0075
*Ccl11*	C-C motif chemokine ligand 11	2.06	0.0067
*F3*	coagulation factor III, tissue factor	2.04	0.000027
*Wnt2*	Wnt family member 2	2.30	0.0049
**BP: Complement activation, *p* = 1.90 × 10^−5^**			
*C6*	complement C6	2.05	0.0073
*Cfh*	complement factor H	1.84	0.025
*C7*	complement C7	0.0001	0.030
**BP: Cellular response to interferon-gamma, *p* = 4.34 × 10^−4^**			
*Ccl11*	C-C motif chemokine ligand 11	2.06	0.0067
*Mrc1*	mannose receptor, C type 1	1.59	0.029
*Ccl6*	chemokine (C-C motif) ligand 6	2.12	0.0056
*Cfh*	complement factor H	1.84	0.025
**BP: Cellular response to interleukin-1, *p* = 5.84 × 10^−4^**			
*Smpd3*	sphingomyelin phosphodiesterase 3	2.35	0.0049
*Ccl11*	C-C motif chemokine ligand 11	2.06	0.0067
*Fn1*	fibronectin 1	2.59	0.00051
*Ccl6*	chemokine (C-C motif) ligand 6	2.12	0.0056
**BP: Chemokine-mediated signaling pathway, *p* = 7.25 × 10^−4^**			
*Ccr1*	C-C motif chemokine receptor 1	2.19	0.0075
*Ccl11*	C-C motif chemokine ligand 11	2.06	0.0067
*Ccl6*	chemokine (C-C motif) ligand 6	2.12	0.0056
**BP: Chronic inflammatory response, *p* = 7.28 × 10^−4^**			
*Ccl11*	C-C motif chemokine ligand 11	2.06	0.0067
*Vnn1*	vanin 1	2.10	0.0018

**Table 4:** Differentially expressed genes from biological processes (BP) identified by Gene set enrichment analysis (GSEA) in SHR rats treated with 1% NaCl drinking solution versus control SHR rats drinking tap water.

Symbol	Name	logFC	Adjusted *p* Value
**BP: Keratinocyte differentiation, epidermis development, *p* = 1.69 × 10^−7^**			
*Lc1m*	late cornified envelope 1M	1.75	0.025
*Lce1f*	late cornified envelope 1F	1.62	0.036
*Krt1*	keratin 1	2.76	0.000003
*Krt10*	keratin 10	2.98	0.000013
*Lce1l*	late cornified envelope 1L	2.29	0.00015
*Krtdap*	keratinocyte differentiation associated protein	1.32	0.040
**BP: Negative regulation of peptidase activity, *p* = 2.85 × 10^−4^**			
*Serpinb3a*	serine (or cysteine) peptidase inhibitor	4.24	0.000027
*Spli*	secretory leukocyte peptidase inhibitor	1.02	0.036
*Slpil3*	antileukoproteinase-like 3	3.02	0.0015
**BP: Neutrophil chemotaxis, *p* = 2.77 × 10^−6^**			
*Il1b*	interleukin 1, beta	1.93	0.011
*Il36g*	interleukin 36, gamma	2.50	0.0024
*Il36rn*	interleukin 36 receptor antagonist	1.45	0.040
*Cxcr2*	C-X-C motif chemokine receptor 2	1.56	0.013
*Ccl22*	C-C motif chemokine ligand 22	1.87	0.0072
*Il36b*	interleukin 36, beta	1.81	0.031
**BP: Establishment of skin barrier, *p* = 1.83 × 10^−4^**			
*Alox12b*	arachidonate 12-lipoxygenase, 12R type	2.43	0.0028
*Krt1*	keratin 1	2.76	0.000003
*Flg2*	filaggrin family member 2	2.87	0.00002
**BP: Negative regulation of endopeptidase activity, *p* = 6.44 × 10^−6^**			
*Serpinb12*	serpin family B member 12	2.98	0.000004
*Stfa3*	stefin A3	2.99	2.89e-10
*Serpina12*	serpin family A member 12	2.12	0.0029
*Stfa2*	stefin A2	2.47	0.00017
**BP: Regulation of signaling receptor activity, *p* = 9.08 × 10^−4^**			
*Btc*	betacellulin	3.39	0.00058
*Il1b*	interleukin 1, beta	1.93	0.011
*Il36g*	interleukin 36, gamma	2.50	0.0024
*Il36rn*	interleukin 36 receptor antagonist	1.45	0.040
*Il18*	interleukin 18	1.96	0.013
*Ccl22*	C-C motif chemokine ligand 22	1.87	0.0072
*Il36b*	interleukin 36, beta	1.81	0.031

## Data Availability

Gene expression data are available in ArrayExpress database (accession number E-MTAB-11355).

## Supplementary Materials

The following supporting information can be downloaded at: https://www.mdpi.com/article/10.3390/biomedicines10020376/s1, Figure S1: Principal component analysis based on expression of 500 genes with highest variance.
