# Supplementary material for: Sodium Accumulation and Blood Capillary Rarefaction in the Skin Predispose Spontaneously Hypertensive Rats to Salt Sensitive Hypertension
Source: Biomedicines. 2022 Feb 4;10(2):376. doi: 10.3390/biomedicines10020376 (PMC8962406; doi:10.3390/biomedicines10020376)
Supplement: Supplementary file 1 [file biomedicines-10-00376-s001.zip › biomedicines-1574135-supplementary.pdf]

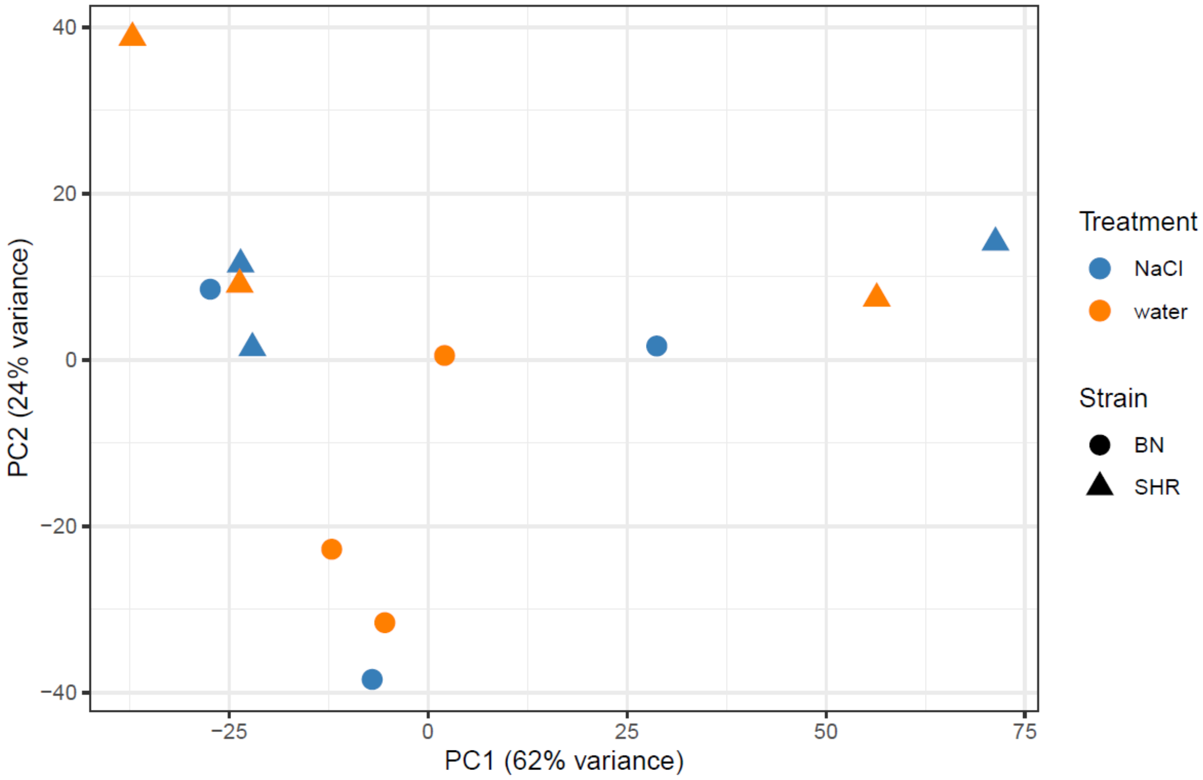

Figure S1. Principal component analysis (PCA) based on expression of 500 genes with highest variance. Percentage in axis labels represents ratio of total variance explained.
